# Effect of components on the curing of glycidyl azide polymer spherical propellant through rheological method

**DOI:** 10.1098/rsos.181282

**Published:** 2018-10-24

**Authors:** Liming He, Wei He, Zhongliang Ma

**Affiliations:** School of Environment and Safety Engineering, North University of China, Taiyuan 030051, People's Republic of China

**Keywords:** GAP spherical propellant, energy components, isoconversional method, rheological kinetics

## Abstract

We have conducted a novel study of the influence of energy components (RDX, AP and CL-20) on curing kinetics of glycidyl azide polymer (GAP) spherical propellant based on rheological method. The autocatalytic model was used to describe curing kinetics and the parameters were determined by the model-fitting method. It was found that the incorporation of components hinders the cross-linking reaction of GAP spherical propellant. Integral isoconversional method was used on rheological kinetics to investigate the changes of the activation energy and we confirmed that the incorporation of components increased the activation energy. It was also found that such components had no effect on the trend of activation energy curves but shrank the peak value at *a* = 0.2. Dynamic mechanical analysis (DMA) showed the differences between pure curing system and its components. These findings are potentially helpful to control the curing effectively and optimize the processing schedules. The addition of components decreased *α* translation temperature which means the reduction in cross-links. The differences in the values of loss factor tan *δ* and *β* translation showed that pure curing system has lower resistance for side chain to motion.

## 1. Introduction

**Figure 1.**
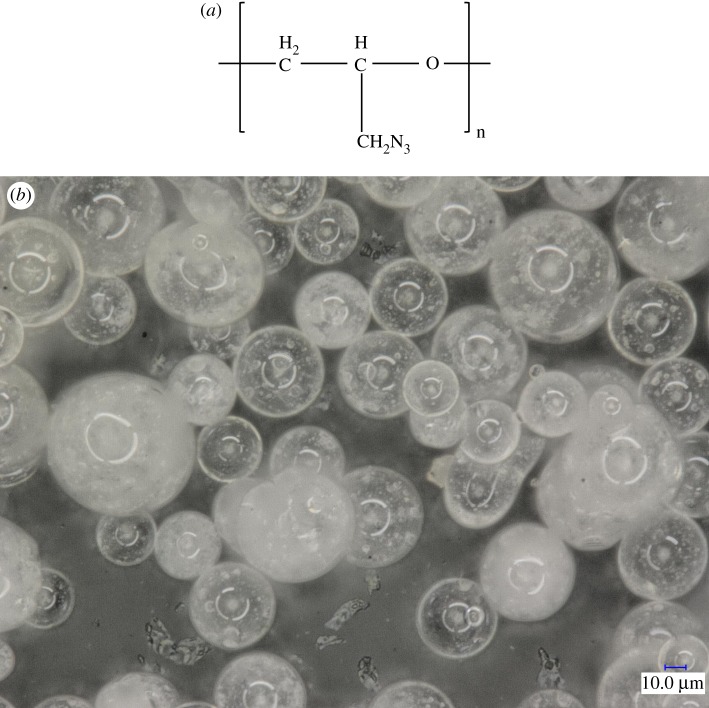
The structure of GAP (*a*) and SEM image of GAP spherical propellant (GAP : nitrocellulose = 1 : 4) (*b*).

Glycidyl azide polymer (GAP) is a binder which is widely used in propellant [1–6]. GAP spherical propellant prepared by an internal solution method is one of the most popular components of composite modified double-base (CMDB) propellant [7,8]. The structure of GAP and the SEM image of GAP spherical propellant are shown in figure 1. This spherical propellant, overcoming the limited mechanical properties of traditional GAP propellant, possessing high enthalpy and density, non-corrosive, low characteristic signal and low sensitivity of the excellent performance, had been widely used in solid propellants and gelled propellants [9–11].

Rocket propellants, with a high specific impulse, commonly contain ammonium perchlorate (AP), hexanitrohexaazaisowurtzitane (CL-20), hexahydro-1,3,5-trinitro-1,3,5-triazine (RDX) and other energetic materials. The sensitivity, thermal characterization, thermal stability and mechanical properties of such components have been widely investigated [12–15]. However, previous studies hardly focused on the influence of components on the curing processes. Few reports have been found on the cure kinetics of GAP [16–18], but the influence of components have not been mentioned either.

Curing process is crucial for solid propellant, because its mechanical properties rely on the chemical structure of the resin and the three-dimensional polymer network achieved by curing. In order to improve the mechanical properties of solid propellant, a deep understanding of curing mechanisms and cure kinetics is the key. Thus, one can obtain an optimal cure and model the reaction processes as accurately as possible [19,20]. The addition of components is believed to have influences on the curing of propellant, but few works have been done to investigate how these components influence the curing processes. For GAP spherical propellant, the components would have a more heavy effect on the curing processes because of its high viscosity. Therefore, it is important to understand the roles of such components to optimize the processing schedules and the properties of the final product.

As for its high viscosity mentioned before, processing of these systems warrants proper knowledge of the rheological behaviour of the blend during the curing process. In this work, we used the rheological method to investigate the cure kinetics of GAP spherical propellant which has been successfully used in other works [7,21,22]. As the network can be formed by chemical reaction between functional group on linear chains to form a three-dimensional polymer network structure and by different kinds of physical forces, such as hydrogen bonds, electrostatic attraction and van der Waals forces, rheological methods are useful for monitoring the curing process, overcoming the limitations that conventional methods cannot investigate the changes in physical conditions.

Glass transition temperature (*T*_g_) is the property that dictates the potential application of a given resin as a function of the molecular architecture and depends on different parameters such as the functionality of the hardener, the conversion degree, the curing cycle, etc. Given the importance of the glass transition temperature in view of its link with mechanical properties, it is necessary to provide the information about *T*_g_. In this work, dynamic mechanical analysis (DMA) has been used to investigate the glass transition temperature of different curing systems. The data of DMA has been used to compare the effect of different components on curing process.

## 2. Material and methods

### 2.1. Material

GAP polyol was received from Hubei Aviation Institution of Chemical Technology. It is diol with average molar weights of 5770 g mol^−1^. *N*-butyl-*N*-(2-nitroxy-ethyl) nitramine (BuNENA) was supplied by Luoyang Li Ming Chemical Industry Institution. Catalyst (T_12_) and isophorone diisocyanate (IPDI) were obtained from Shanghai Taizheng Chemical Company. RDX, CL-20 and AP were received from Beijing Institute of Technology. GAP spherical propellant (GAP : nitrocellulose = 20 : 80) was made by ourselves and it contains nitrocellulose which is a poly-functional polymer and acts as a chain extender.

### 2.2. Sample preparation

The first stage of the procedure was to dry materials at 50°C for 10 h; specially, BuNENA was baked under reduced pressure at 35°C for 5 h. After mixing the BuNENA and GAP spherical propellant in 1 : 1 mass proportions, the mixture was placed in a dryer for 5 h to dissolve spherical propellant. Then, 10% high energy component, 0.2% IPDI (R = 1) and 0.002% catalyst were added, and the mixture was stirred for 30 min before testing.

### 2.3. Rheological measurements

Storage modulus, loss modulus and complex viscosity of the GAP spherical propellant systems were studied by Anton Paar Physica MCR 302 rheometer with parallel plate tools of 25 mm diameter, and the gap height between parallel plates was set at 1 mm. Isothermal measurements were carried out in 50–70°C temperature range, with an angular frequency of 1 Hz and an initial strain of 1%.

### 2.4. DMA measurement

The cured GAP spherical propellant samples were tested on a dynamic mechanical analyser (DMA, Q800, TA) by single-cantilever mode. The temperature ranged from −80 to 100°C. The frequencies were set at 1 Hz, with oscillation amplitude at 15 mm and heating rate was fixed at 3°C min^−1^. The experimental data were analysed with TA Universal 2000 software.

## 3. Results and discussion

### 3.1. Fundamental theory on curing reaction kinetics

The curing process is related to the dynamic rheological parameters, such as storage modulus *G′*, loss modulus *G″* and complex viscosity η*. For isothermal rheological tests, the degree of conversion, *α*, can be described by *G*′. The relation is given as:

3.1 $$a=\frac{{G^{\prime }}_{t}-{G^{\prime }}_{0}}{{G^{\prime }}_{\infty }-{G^{\prime }}_{0}},$$

where $G_{\infty }^{\prime }$ is the value of *G*′ at the end of the curing reaction. This value is proportional to the maximum cross-linking density of the network reached under given curing conditions. $G_{0}^{\prime }$ is the storage modulus at the beginning of the reaction, and $G_{t}^{\prime }$ is the measured dynamic storage modulus as a function of time.

The fundamental rate equation which describes the reaction rate as a function of time and temperature is

3.2 $$\frac{da}{dt}=k(T)f(a),$$

where *t* is the time, *k*(*T*) is the rate constant which can be replaced by Arrhenius equation (equation (3.3)) and *f*(*a*) is the model function that depends on the reaction mechanism.

3.3 $$k(T)=A\;\exp\left(-\frac{E_{a}}{RT}\right),$$

where *A* is the pre-exponential factor, and *E*_a_ is the apparent activation energy of the process.

### 3.2. Model-fitting kinetics

**Figure 2.**
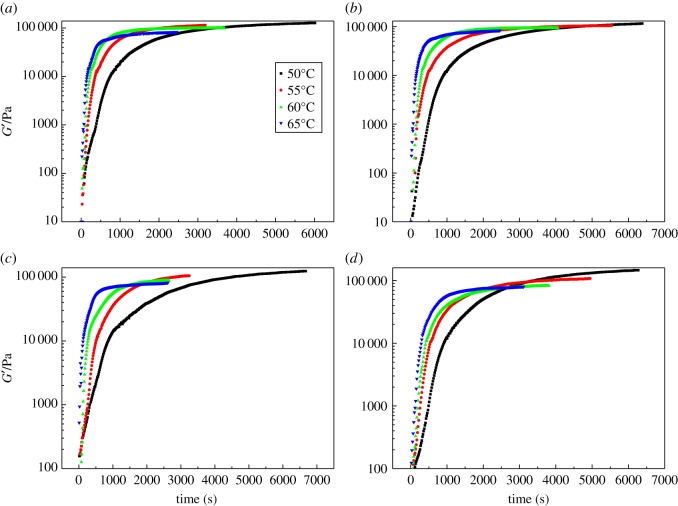
Rheology isothermal curves of different curing systems at 50°C, 55°C, 60°C and 65°C. (*a*) IPDI/GAP spherical propellant (pure curing system); (*b*) 0.1CL-20 curing system; (*c*) 0.1AP curing system; (*d*) 0.1RDX curing system. The raw data of (*a–d*) can be found in the electronic supplementary material.

Figure 2 shows the relationship between storage modulus *G′* and time of rheological tests for the curing reaction. It can be seen that storage modulus after curing $\left(G_{\infty }^{\prime }\right)$ was almost the same for each curing system at a given temperature, but became a little higher when the temperature dropped. The reason can be the higher temperature enhanced the mobility of molecules but it was believed to have no impact on the curing mechanism.

**Figure 3.**
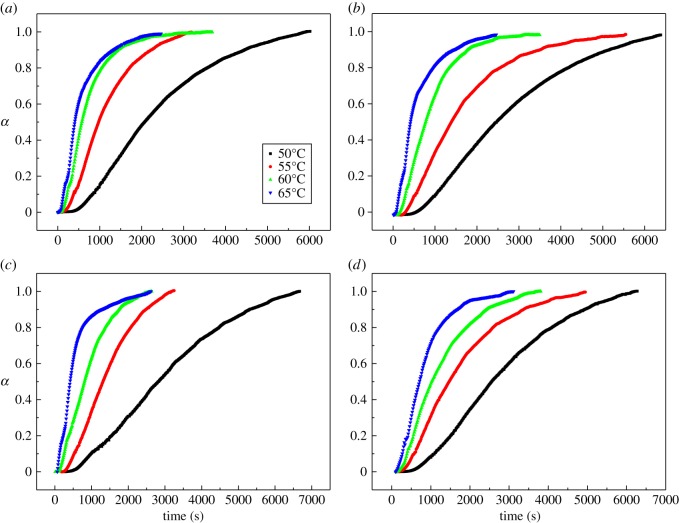
Evolution of conversion degree with temperature during the curing process. (*a*) Pure curing system; (*b*) 0.1CL-20 curing system; (*c*) 0.1AP curing system; (*d*) 0.1RDX curing system.

According to equation (3.1), the change of curing degree with different curing temperatures and time can be investigated (figure 3). The curves in figure 3 showed a rapid increase during the initial reaction stage and then slowed down until they reached a certain value. The rapid increase of degree in the initial reaction stage was attributed to the chain extension and cross-linking of the molecular chain, and these reactions reduced the mobility of the reacting molecules and slowed down the conversion rate.

**Figure 4.**
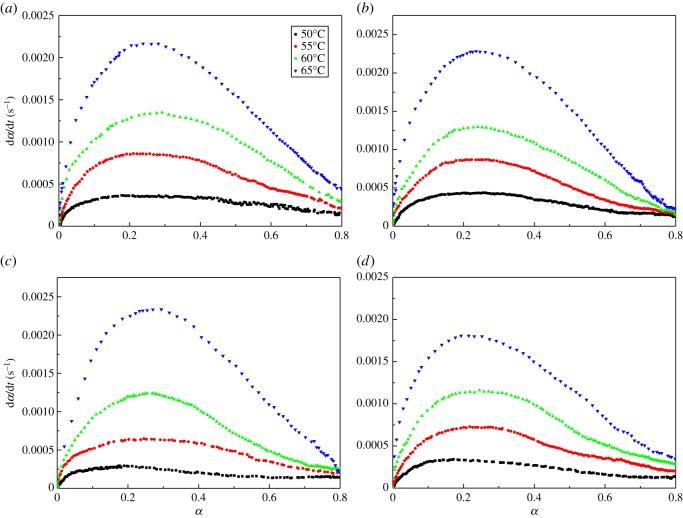
Variation of the conversion rate in the conversions of GAP spherical propellant by isothermal rheological experiments. (*a*) Pure curing system; (*b*) 0.1CL-20 curing system; (*c*) 0.1AP curing system; (*d*) 0.1RDX curing system.

As we can see from figures 2 and 3, no significant difference can be directly found. The pure curing system (i.e. free of high energy components) and other curing systems with high energy components have a similar trend in both storage and conversion curves. In order to compare the details of different curing systems, the relationship between the conversion rate and conversion are shown in figure 4. Initially, the reaction rate increased with time, and then it gradually decreased to zero. The conversion rate had a maximum value at an intermediate curing degree, which indicated that all testing system showed autocatalytic characteristic [23,24]. It may be that the resulting carbamate group (–NH–CO–O) catalyses the urethane esterification of –NCO with –OH and causes the reaction to exhibit autocatalysis [25].

For the calculation of the kinetic parameters, an autocatalytic model was employed for the analysis of the cure of GAP special propellant and its components [24]. An empirical rate equation proposed by Kamal can be applied to thermosetting cure that shows autocatalytic behaviour.

3.4 $$\frac{da}{dt}=(k_{1}+k_{2}a^{m}){\left(1-a\right)}^{n},$$

where *k*_1_ is the non-autocatalytic rate constant, *k*_2_ is the autocatalytic reaction rate constant associated with the conversion rate, while *m* and *n* are exponents of the empirical equation. Both *k_1_* and *k_2_* follow the Arrhenius equation.

According to equaiton (3.4), the parameters *k*_1_, *k*_2_, *m*, *n* and *A* can be determined. Table 1 lists the results of kinetic analysis based on the autocatalytic model.

**Table 1.** The values of kinetic parameters according to equation (3.4).

	*T* (°C)	*k*_1_ (s^−1^)	*k*_2_ (s^−1^)	*m*	*n*	Ln *A*_1_	Ln *A*_2_	*E*_1_ (KJ mol^−1^)	*E*_2_ (KJ mol^−1^)
pure	50	0.000032	0.00114	0.38	1.27	44.37	37.48	146.41	116.87
	55	0.000118	0.0024	0.35	1.41				
	60	0.000241	0.00413	0.47	1.49				
	65	0.000367	0.00842	0.53	1.51				
CL-20	50	0.000035	0.00131	0.37	1.45	45.35	41.74	148.98	127.85
	55	0.000121	0.00271	0.41	1.54				
	60	0.000201	0.00487	0.44	1.63				
	65	0.000453	0.0106	0.51	1.70				
AP	50	0.00003	0.00129	0.37	1.33	44.85	38.78	147.82	122.33
	55	0.000127	0.00187	0.42	1.38				
	60	0.000192	0.00513	0.41	1.43				
	65	0.000391	0.00910	0.52	1.56				
RDX	50	0.00004	0.00109	0.47	1.06	45.32	45.17	148.63	129.20
	55	0.000133	0.0019	0.39	1.37				
	60	0.000224	0.00357	0.42	1.47				
	65	0.000512	0.00949	0.52	1.53				

From table 1, we can see that autocatalytic reaction constant was much higher than non-autocatalytic reaction constant at a given temperature, and the rate constant increased with curing temperature. As equation (3.3) shows that both *k*_1_ and *k*_2_ follow the Arrhenius law, the activation energy for the non-autocatalytic and autocatalytic reactions can be determined by the slope of the linear fit line of ln *k* against 1/*T*.

**Figure 5.**
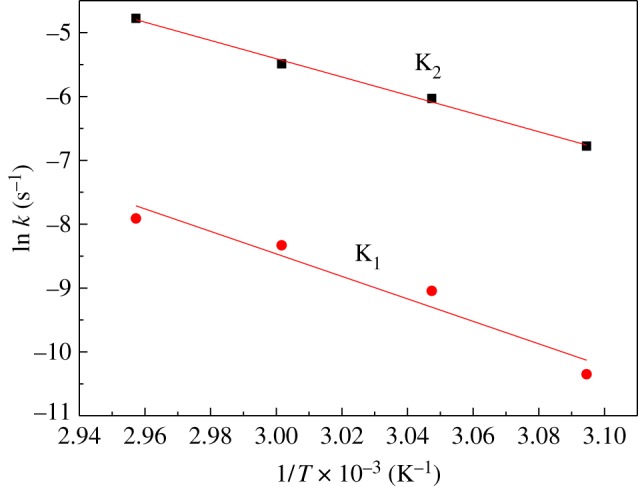
Arrhenius plots of ln *k* against 1/*T* to derive the values of the activation energies for pure curing system.

Figure 5 depicts the plots of the ln *k* against 1/*T* for pure curing system, from which a linear correlation can be discovered. In the same way, the activation energies of other curing systems can be discovered (table 1). It can be found that all kinds of testing components increased the activation energy and decreased the pre-exponential factors of pure curing system for both non-autocatalytic and autocatalytic reactions. This result revealed that the incorporation of components (CL-20, AP or RDX) hindered the cross-linking reaction of IPDI/GAP spherical propellant. It can also be seen that RDX had larger activation energy values than the other components. The reason might be that the addition of such component increases the viscosity of the curing system because of its smaller particle size, which has a significant influence on the mobility of molecules. Furthermore, stronger hydrogen bonds and van der Waals interaction exist between RDX and GAP [26], which further increases the resistance of the reaction.

### 3.3. Integral isoconversional method

Unlike the model-fitting method, the isoconversional methods take their origin in the isoconversional principle that the reaction rate at the constant extent of conversion is only a function of temperature, which can be expressed as equation (3.5):

3.5 $${\left[\frac{\partial \ln(da/dt)}{\partial T^{-1}}\right]}_{a}={\left[\frac{\partial \ln k(T)}{\partial T^{-1}}\right]}_{a}+{\left[\frac{\partial \ln f\;(a)}{\partial T^{-1}}\right]}_{a}.$$

The subscript *a* indicates the certain conversion corresponding with the kinetic parameters. Because at *a* = constant, *f(a)* is a constant, and the second term in the right-hand side of equation (3.5) is zero, thus:

3.6 $${\left[\frac{\partial \ln(da/dt)}{\partial T^{-1}}\right]}_{a}=-\frac{E_{a}}{R}.$$

The relationship between activation energy and conversion can be established by equation (3.6). However, direct derivation of this method will lead to serious error. The advanced isoconversional method proposed by Vyazovkin *et al*. [23] can eliminate the error by performing the integration over small segments of either temperature or time. This method can be applied to any temperature programmes that are following the equations:

3.7 $$\Phi (E_{a})=\sum_{i=1}^{n}\sum_{\;j\neq i}^{n}\frac{J[E_{a},T_{i}(t_{a})]}{J[E_{a},T_{j}(t_{a})]}$$

and

3.8 $$J(E_{a},T_{a})=\int_{t_{a}-\Delta a}^{t_{a}}\exp\left[\frac{-E_{a}}{RT(t)}\right]\;dt,$$

where *i* and *j* denote the different thermal experiments conducted under various temperatures, Δ*a* is the tiny increment in *a*. *t_a_* is the reaction time during which *a* is reached. The *E_a_* value is determined by minimizing equation (3.7) for a series runs conducted under different temperature programmes.

**Figure 6.**
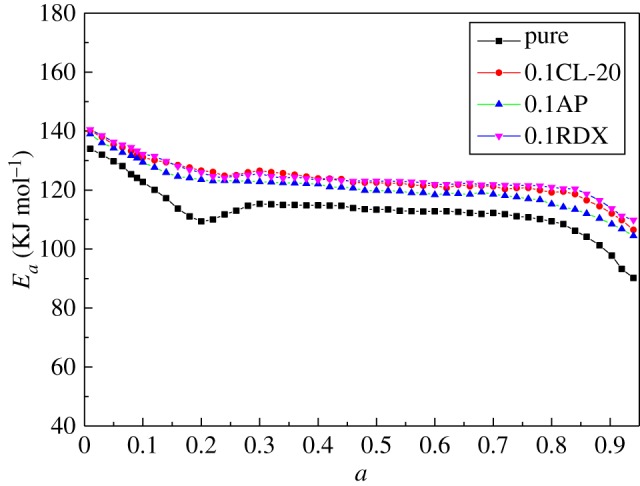
Activation energy *E_a_* as a function of conversion.

Figure 6 shows the activation energy calculated by equations (3.7) and (3.8) for pure curing system and components. It can be observed that *E_a_* has initial high values, following a gradual reduction to about 0.8 conversion degree and rapidly decreases when *a* exceeds 0.8. At the beginning stage, the system requires a contribution of energy to start the cure. Once the reaction has started, activation energy needed for the further reaction decreases. At the point of *a* = 0.8, diffusion control is dominated. Thus, the further reaction can only rely on short-range motions of adjacent groups and activation energy decreases rapidly.

It also can be seen that pure curing system had a lower activation energy than its components and a concave line can be observed at *a* = 0.2. For curing systems incorporated with components, the trends of activation energy curves had no significant difference with pure curing system except the shrink on the peak value at *a* = 0.2, which means the incorporation of those energetic materials had no side reactions but changed the resistance of reaction. The different values of activation energy curves indicated that components increased the activation energy for more energy need to overcome the resistance of components which conformed to the model-fitting results. However, no obvious difference can be observed from activation energy curves for components, especially for CL-20 and RDX.

Integral isoconversional method had been first used in rheological kinetics. It can be seen that integral isoconversional method was more accurate and showed more details of apparent activation energy than conventional methods. It is useful not only in DSC methods but also in rheological methods.

### 3.4. DMA analysis

**Figure 7.**
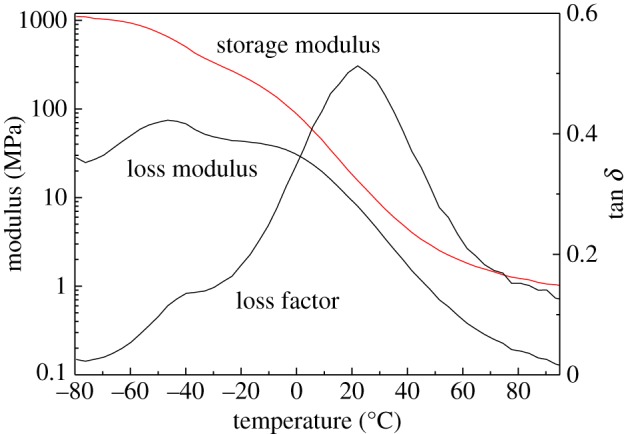
Dynamic mechanical temperature spectra of cured system.

**Figure 8.**
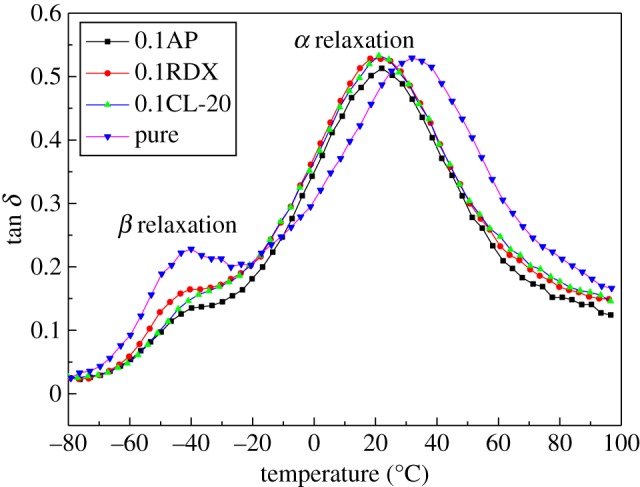
*β* and *α* relaxation processes for different curing systems.

DMA technology is one of the commonly used characterization methods for studying the structure of materials and their chemical and physical properties. It has become one of the important methods for studying the properties of polymer materials. The typical dynamic modulus and loss factor tan *δ* as a function of temperature under the frequency of 1 Hz are demonstrated in figure 7. The peak temperature of tan *δ* was taken as the translation temperature at which relaxation occurs. Figure 8 compares the influence of different components on the *β* and *α* translation temperature in different curing systems, and the values of *T_β_* and *T_α_*_,_ loss factors (tan *δ_β_* and tan *δ_α_*) are shown in table 2.

**Table 2.** Relation temperatures of loss factors for *β* and *α* relaxation processes in different curing systems.

	*T_β_* (°C)	*T*_α_ (°C)	tan *δ_β_*	tan *δ_α_*
pure	−40.39	33.64	0.23	0.54
CL-20	−38.30	20.29	0.15	0.52
AP	−39.64	22.39	0.13	0.51
RDX	−40.22	18.90	0.16	0.52

It can be seen clearly that all curves had a similar *β* translation temperature, but the value of tan *δ_β_* for pure curing system was much higher. The reason was that pure curing system had lower resistance for side chain to motion. As for *α* translation temperature, which was considered as the reflection of molecular architecture after curing, the pure curing system had a much higher *α* translation temperature than other curing systems. It indicated the incorporation of such components reduced the cross-links of IPDI/GAP spherical propellant curing system.

## 4. Conclusion

The effect of components on the curing of GAP spherical propellant has been tested by the rheological method. The reaction showed a typical feature of autocatalytic reaction. The activation energies of IPDI/GAP spherical propellant and incorporation of different energetic materials (CL-20, AP or RDX) has been investigated by model-fitting and integral isoconversional methods. It has been found *E_a_* has initial high values, following a gradual reduction, and such components reduced the cross-links of IPDI/GAP spherical propellant curing system, while no side reaction had been found. Comparing the difference between RDX and other components, one can find that RDX has the biggest influence on the curing of GAP spherical propellant.

The results here presented provide a novel contribution to the understanding of the effect of the components on the curing of IPDI/GAP spherical propellant, which is useful to control the curing effectively and to optimize the processing schedules. The results would benefit the further study on the GAP spherical propellant and the improvement of mechanical properties of such solid propellant.

## Supplementary Material

Raw data in text format

## Data accessibility

Raw data can be found in the electronic supplementary material. All files are text formats recording raw data of four curing system (A: IPDI/GAP spherical propellant (pure curing system); B: 0.1CL-20 curing system; C: 0.1AP curing system; D: 0.1RDX curing system) at 50, 55, 60 and 65°C.
